# Parathyroid autotransplantation at a novel site for better evaluation of the grafted gland function: study protocol for a prospective, randomized controlled trial

**DOI:** 10.1186/s13063-019-3195-9

**Published:** 2019-01-31

**Authors:** Qiuxia Cui, Deguang Kong, Zhihua Li, Kun Wang, Dan Zhang, Jianing Tang, Xing Liao, Qianqian Yuan, Yan Gong, Gaosong Wu

**Affiliations:** 1 Department of Thyroid and Breast Surgery, Zhongnan Hospital, Wuhan University, 169 Donghu Road, Wuhan, Hubei 430071 China; 2 Department of General Surgery, Hubei Provincial Hospital of TCM, 856 Luoyu Road, Wuhan, China; 3 Department of Thyroid and Breast Surgery, Tongji Hospital of Tongji Medical College, Huazhong University of Science and Technology, 1095 Jiefang Avenue, Wuhan, China; 4 Department of Biological Repositories, Zhongnan Hospital, Wuhan University, 169 Donghu Road, Wuhan, Hubei 430071 China

**Keywords:** Parathyroid autotransplantation, Novel site, Assessing method, Parathyroid hormone

## Abstract

**Background:**

Hypoparathyroidism is one of the most common complications encountered in thyroidectomy. In addition to parathyroid in-situ preservation, parathyroid autotransplantation (PA) is another important remedial method for patients whose parathyroid glands have been removed. However, an accurate evaluation method for the function of a transplanted parathyroid is lacking. Our preliminary study indicated that patients with PA at novel sites near antecubital veins had higher serum concentrations of parathyroid hormone (PTH). Therefore, the main hypothesis is that a grafted site closer to the cephalic vein is more useful for better evaluation of transplanted parathyroid function. This study aims to confirm the more efficient and accurate evaluation system through a prospective, randomized controlled trial.

**Methods:**

In total, 280 patients will be enrolled in this study and randomly divided into two groups: 140 patients with transplanted parathyroid glands in the traditional sites (group A) and the other 140 transplanted in the novel sites (group B), close to the antecubital veins. The serum concentration of PTH and calcium ion from both forearms will be measured and monitored regularly for 12 months. The primary outcome of this trial will be the survival of grafted glands, defined as the ratio of PTH between the grafted vs. the non-grafted forearms being no less than 1.5. The secondary outcome is hypoparathyroidism, defined as the PTH level from the non-grafted forearms being less than 15 pg/ml (normal range 15–65 pg/ml).

**Discussion:**

Our results from this study should provide a more accurate method to evaluate the function of transplanted parathyroid glands by comparing PTH concentrations in both the grafted and non-grafted forearms following PA at novel sites. A better PTH measurement is helpful not only for the management of postoperative patients, but also for further identification of factors affecting PA success.

**Trial registration:**

ClinicalTrials.gov, ID: NCT02906748. Registered on 16 March 2016.

**Electronic supplementary material:**

The online version of this article (10.1186/s13063-019-3195-9) contains supplementary material, which is available to authorized users.

## Background

Parathyroid autotransplantation (PA) is an important technique for dealing with parathyroid glands removed in thyroidectomy and other neck surgeries [1, 2]. Unintentional parathyroidectomy is a major cause of postoperative hypoparathyroidism in total thyroidectomy with or without lymph node dissection [3, 4]. The incidence of unintentional parathyroidectomy during thyroidectomy has been shown to be 9–19% [5]. It has been reported that the number of remaining parathyroid glands in situ is a predictive variable for the rate of permanent hypoparathyroidism [6]. Although most parathyroid glands were preserved in situ in this study, permanent hypoparathyroidism was still observed frequently [6]. The incidence of permanent hypoparathyroidism could be up to 14.3% [7, 8] after thyroidectomy. PA has been reported to reduce the rate of permanent hypoparathyroidism [8, 9].

Our previous studies, recruiting 218 patients with thyroidectomy [10], indicated that approximately 17.5% of patients needed the partial tissue of one parathyroid gland for autotransplantation, and that 6.6% needed one or more integral parathyroid gland(s). However, there was no widely accepted, accurate method to evaluate the transplanted parathyroid glands. The currently popular method is to combine clinical symptoms and parathyroid hormone (PTH) serum concentration. However, this method cannot distinguish between the function of the remaining and transplanted parathyroid glands.

The PTH-gradient method, which compares serum PTH concentrations between the grafted and the non-grafted forearms, was originally applied to examine the function of transplanted parathyroid glands in patients with hyperparathyroidism [11]. A ratio of PTH from the grafted to non-grafted forearms of no less than 1.5 was considered to confirm successful PA [12]. It was mostly applied to monitor PTH in hyperparathyroidism patients with parathyroidectomy after PA [13, 14]. Rothmund et al. reported that the survival rate of PA with hyperparathyroidism was 31% (20 patients) [15], and Anamaterou et al. suggested that the survival rate was 45% in 19 patients using the ischemic-blockage Casanova test [16]. There are few reports regarding randomized controlled trials to evaluate the function of transplanted parathyroid glands using this PTH-gradient method.

In the previous study of PA in thyroidectomy, the grafted site in the forearm strategy was applied to several patients who had been examined and found to have a higher concentration of serum PTH from the closer-to-antecubital-vein graft sites than the more distant ones. To promote the accuracy of this PTH-gradient method to evaluate PA success for thyroidectomy patients after autotransplantation with normal parathyroid glands, we hypothesized that PA at a novel site closer to the antecubital veins could result in a higher concentration of serum PTH and a more accurate ratio of PTH from the grafted forearm relative to the non-grafted one. Vice versa, the null hypothesis was that there was no significant difference of either serum concentration or ratio of PTH from both forearms. This prospective, randomized controlled trial aims to establish a modified PTH-gradient method by PA at the novel sites to promote the accuracy of transplanted parathyroid gland evaluation. Our results should contribute to a more accurate evaluation of transplanted parathyroid function in PA patients, and facilitate further investigation of potential factors affecting the survival of transplanted parathyroid glands.

## Methods

### Study design

This is a prospective, randomized controlled trial to apply PA at the novel sites and to assess the survival rate of transplanted parathyroid glands in thyroidectomy patients. A 1:1 block randomization will be performed at the same clinical center and operations will be carried out by the same experienced surgeon in Tongji Hospital (Wuhan, China). The study has been approved by the Ethics Committee of Tongji Hospital, Tongji Medical College, Huazhong University of Technology Institutional Review Board with approval given on 25 June 2015 (reference number: TJ-C2015602) (Additional file 1).

In this single-center study, hospitalized patients with primary thyroidectomy in Tongji Hospital will be enrolled. Stochastic indicators were used to generate the random allocation sequence of the patients. Sequentially numbered containers were employed to implement the random allocation sequence and to conceal the sequence until interventions are assigned for implementation. Analysts in our study generated the random allocation sequence while care providers numbered the qualified participants. The surgeon assigned participants to interventions based on the condition of their parathyroid glands during thyroidectomy. All subjects, researchers in the laboratory and data analysts will be blind to the group classification.

Primary endpoint: the ratio of the serum PTH level from the grafted vs. the non-grafted forearm level is no less than 1.5 and the percentages of the 1.5 ratio in the experimental group are obviously higher than those in the control group. Since successful PA was defined as the ratio of the PTH level from the grafted to the non-grafted forearms as not less than 1.5, we set the primary endpoint in line with expert opinion, and the percentages of no less than the 1.5 ratio in the experimental group are obviously higher than those in the control group. 

Secondary endpoints: serum PTH less than 15 pg/ml, and lasting for more than 6 months, as well as a low serum calcium level requiring oral supplements to be taken. Duration of hospital stay will be documented according to hospital charts. During the 1-month to 12-month follow-ups after surgery, patients will be interviewed to assess for postoperative hypocalcemia.

**Fig. 1:**
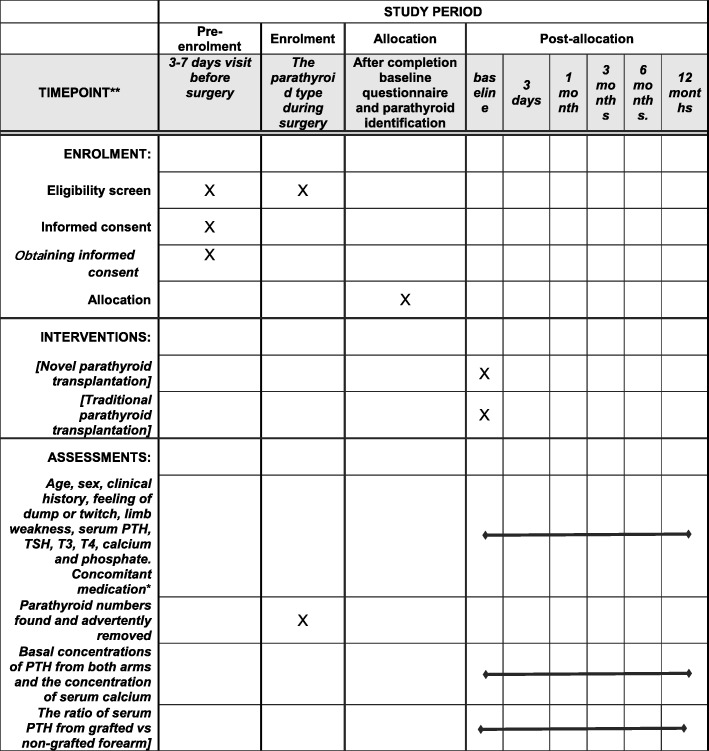
Standard Protocol Items: Recommendations for Interventional Trials (SPIRIT) Figure: the schedule of enrollment, interventions, and assessments

The study was registered at ClinicalTrials.gov and reported according to the Standard Protocol Items: Recommendations for Interventional Trials (SPIRIT) guidelines, including the SPIRIT Figure (Fig. 1) and SPIRIT Checklist (Additional file 2).

The allocation envelope will be opened by the surgeon when conducting the interference. It will not be possible to mask the treatment allocation from the treating surgeon. Nevertheless, measures will be taken to minimize bias:

1. The patients will not be informed regarding whether they will receive the novel site or the traditional site method
2. To minimize detection and selection bias, the exact method of PA will not be documented in the medical records
3. The investigator performing the statistical analyses will be blinded to treatment assignments until the final analysis is completed

### Study participants

**Fig. 2:**
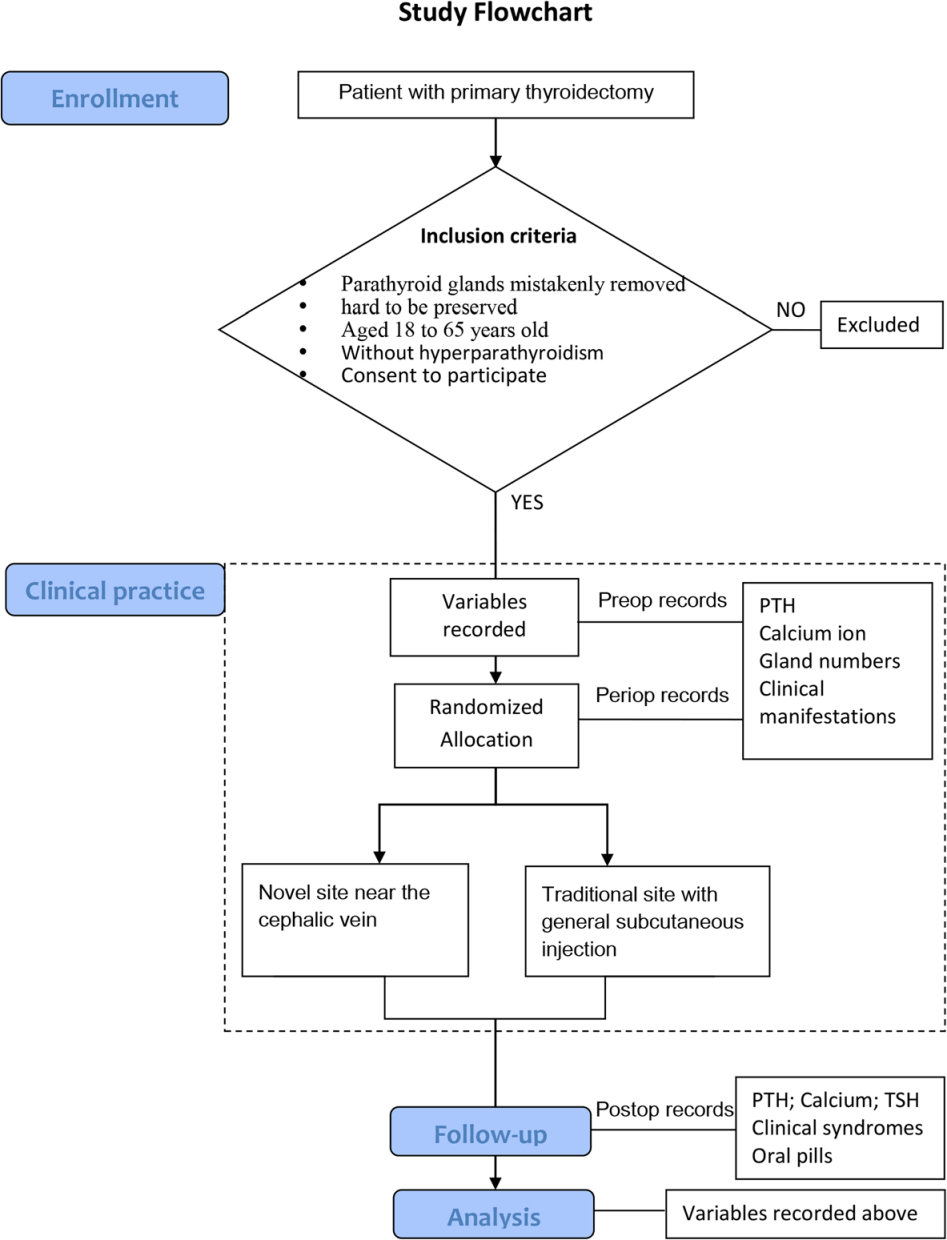
Study flowchart

Those patients with parathyroid glands that are hard to preserve in situ, or that are mistakenly removed during primary thyroidectomy, will be screened for the inclusion criteria in our study, including malignant and non-malignant disease. Since 10–67% of adults have thyroid nodules on ultrasonography, 10–40% of which were reported as malignant [17, 18], both malignant and non-malignant thyroid diseases were taken into consideration at randomization and analysis. Participants aged from 18 to 65 years old will be enrolled, and those with hyperparathyroidism will be excluded (Fig. 2).

### Sample size calculations

The experimental group involves the participants with PA at the novel sites closer to the cephalic vein, while the participants in the control group generally receive subcutaneous transplantion. Since the survival rate of the traditional assessing method was reported as 45% [16], and that of the new method was predicted as 65% in our preliminary study (unpublished data). We set α as 0.05 (bilateral), power as 0.90 and obtained the sample size *N*1 = *N*2 = 128 by PASS 11. To compensate for a loss to follow-up, we set the final sample size as 280 in all.

### Informed consent

Informed consent will be obtained from all participants in the study prior to surgery and confirmation will be made after PA. If the patient is unable to give consent, it will be sought from a close relative. Withdrawal from the study is possible at any time, in accordance with the latest version of the Declaration of Helsinki 2013.

### PA protocol

Patients will undergo operations in the supine position under general anesthesia. The removed parathyroid glands will be placed in sterile physiological salt solution at 4 °C until confirmed by the frozen-section diagnoses. The parathyroid glands will be sliced into 2–4-mm cubes and mixed with 0.5 cc sterile physiological salt solution in a 1-cc syringe. The parathyroid fragments will be subcutaneously injected into the non-dominant forearm with a 16-G needle [19].

**Fig. 3:**
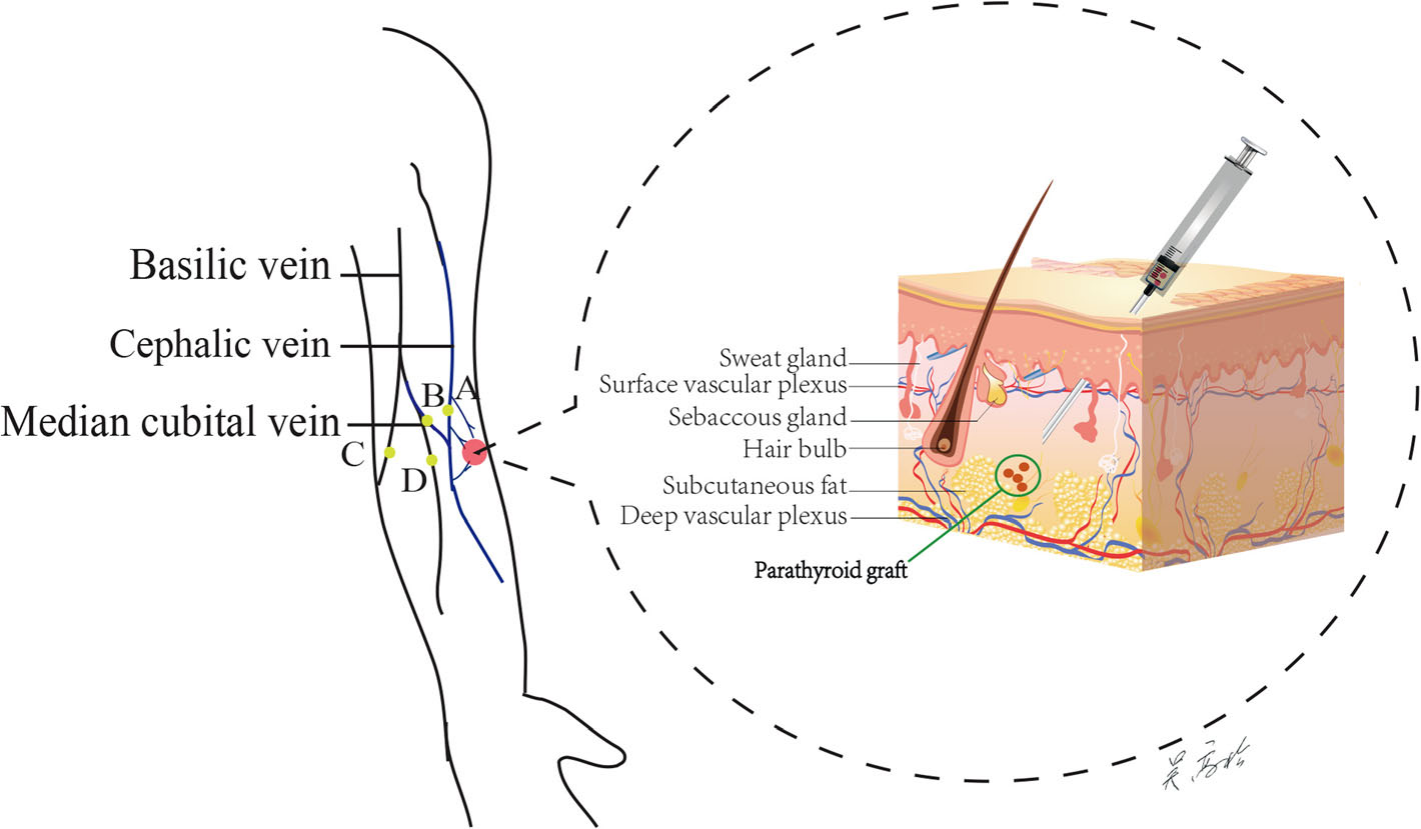
The novel site of parathyroid autotransplantation (PA). In the forearm, the novel site was chosen close to the cephalic vein (point A or B), which is convenient to obtain blood samples. The traditional site is chosen to be more distant to the cephalic vein, such as points C and D

The novel site method means choosing the graft site close to the antecubital veins, including the cephalic vein, the median cubital vein and the basilic vein. During the follow-up, blood samples will be taken from the vein near the graft site (Fig. 3), as well as from the opposite forearm. The traditional site method means general transplantation further away from the relevant antecubital veins. In Fig. 3, the novel sites are points A and B and the traditional are points C and D. All of the grafted forearms from the two groups will be pressed gently immediately after transplantation. The blood samples will be taken at five different time points: 3 days, 1 month, 3 months, 6 months and 12 months after transplantation.

### Baseline variables

Variables will be documented in the Case Report Form (CRF) preoperatively, intraoperatively and within 72 h postoperatively by the attending surgeon.

#### Preoperative variables

These will be age, sex, clinical history, diagnosis, treatment, feeling of dump or twitch, limb weakness, serum PTH concentration, and thyroid-stimulating hormone (TSH), triiodothyronine (T3), thyroxine (T4), calcium and phosphate levels. Concomitant medication will also be recorded for oral supplementation with calcium and calcitriol soft capsules.

#### Intraoperative variables

Unilateral or bilateral surgery, duration of surgery, parathyroid gland numbers found and advertently removed, the color of the parathyroid glands.

#### Postoperative variables

PTH assays taken from the grafted and non-grafted forearms, serum calcium ion and phosphate levels, symptoms of limb numb or twitch, wound healing of the transplantation site, and duration of postoperative drainage.

### Outcome measures

Basal concentrations of PTH from both arms and the concentration of serum calcium will be obtained before and after surgery. Postoperative evaluation of symptoms and signs of hypocalcemia will be recorded as well as oral supplements with calcium and calcitriol. Two researchers will be assigned to record the examination results of participants, and to take blood samples.

### Criteria for discontinuing and quitting the trial

The following participants will quit this study: (1) any participant who develops breast cancer during the follow-up period, as the calcium homeostasis will be interrupted by the required breast cancer chemotherapy and hormone therapy; or (2) if the forearm is too fat to see the exact blood vessel arrangements. In general, no modifying allocated interventions for a given participant will be needed. Adverse events are seldom seen in this study, but sometimes graft-site infection may be observed and will be recorded.

### Follow-up

The follow-up period is 12 months. In order to strengthen the monitoring adherence, most patients will be contacted through WeChat, QQ chat, mobile phones, emails and the regular outpatient interview. Follow-up will be scheduled on the third day and the first, third, sixth and 12th months postoperatively.

### Data management and statistical analysis

One of the researchers, who is an expert in statistics and blind to treatment assignments, will perform the analysis. The sign that a PA has survived will be set as the higher PTH ratio between the grafted and non-grafted forearm, which will be analyzed by a *Χ*^2^ test. All parameters will be analyzed by appropriate statistics with SPSS 22.0, including categorical data and normally distributed numerical data. The former will be compared using a *Χ*^2^ test or Fisher’s exact test and the latter will be analyzed by a *t* test and the Mann-Whitney *U* test if skewed. A Cox model will be used to verify the factors affecting PA survival. Data will be collected in the CRF and consent forms are preserved in a locked computer kept in a locked room. Each participant will be identified by randomization number to ensure the confidentiality of this study.

### Subgroup analysis

We will compare the primary outcome between subgroups characterised by: (1) high vs. low adherence; (2) malignant vs. non-malignant thyroid disease; (3) age categories (18–45, 46+ years); (4) gender and Body Mass Index (BMI) categories (< 25, 25+ kg/m^2^); and (5) parathyroid numbers autotransplanted and preserved.

### Dissemination policy

Trial results will be disseminated to participants through emails, outpatient interviews or social media, including the condition of the transplanted parathyroid glands and the serum levels of PTH and calcium. In particular, those patients developing hypoparathyroidism will be assessed for the number of days needed to adjust their hypocalcemia to a normal level. At the end of the study, we will publish the results as references for health care professionals, the public, and other researchers.

### Data monitoring

An interim analysis will be performed when each group has 140 subjects with a completed 12-month follow-up. The interim analysis will re-evaluate the assumptions made for the study sample size calculation. If the percentages of the 1.5 ratio in the experimental group are obviously higher than those in the control group, the sample size will be re-estimated unless the trial has to be stopped as a result of the interim analysis.

We will perform an interim analysis of each 70 subjects, which focuses on differences of PA survival with different methods. The consents and clinical data will be monitored not only by the supervisor but also by the Data Monitoring Committee in Tongji Hospital, which is independent from this study and has no competing interests.

## Discussion

This is a prospective, randomized controlled trial comparing the evaluation efficiency of the novel site method and the traditional site method of PA in thyroidectomy. The purpose is to investigate whether the novel site close to the cephalic vein could be used to evaluate the function of transplanted parathyroid glands more accurately. Hypoparathyroidism is one of the most common complications in surgeries for thyroid carcinoma [20] because the parathyroid glands are sometimes prone to inadvertent damage, becoming devascularized, or being removed [21]. It has been reported that permanent hypoparathyroidism could be prevented by PA [22]. However, there exists controversy regarding PA efficiency after thyroidectomy [23, 24]. Therefore, it is necessary to establish a more efficient evaluation system for the transplanted parathyroid function.

The novel site for PA might provide a more accurate method to assess PA survival through biochemical measurement of the PTH ratio from the grafted and non-grafted forearms. To test this theory, the current prospective, randomized controlled trial was initiated. This new evaluating system could improve the measurement of the PTH ratio between the grafted and non-grafted forearm and will be convenient for the further study of PA regulation.

The strengths of the study include the prospective, randomized controlled trial design of the novel PA sites combining with the application of the PTH-ratio assessing system. In addition, a digitalized patient chart system ensures easy access, facilitating follow-up.

## Limitations

Although the surgeon could not be blind to the participants’ information, the follow-up information and data analyses will be performed by the researchers blind to the exact PA method. The follow-up of 12 months is also a limitation of the research in postoperative hypoparathyroidism. Besides, the surgical strategy is so homogeneous that subtle differences in the surgical method cannot be excluded. Furthermore, as this novel system is only applied to evaluate PA survival level, the relative factors required for improving PA survival rate will be conducted in the subsequent investigation.

## Trial status

At the time of manuscript submission, the study is in the state of recruiting. The study started in April 2016 and will be completed in December 2018.

### Acknowledegments

The authors acknowledge the support of Tongji Hospital and the relevant Ethical Approval Committee, and the researchers in establishing and monitoring the detailed information of participants. We are grateful for the support from the whole staff involved in thyroid and breast surgery in Tongji Hospital and Zhongnan Hospital.

## Additional files

Additional file 1: Ethic certification of clinical trials. (JPG 184 kb)

Additional file 2: Standard Protocol Items: Recommendations for Interventional Trials (SPIRIT) 2013 Checklist: recommended items to address in a clinical trial protocol and related documents*. (PDF 210 kb)

## Abbreviations

CRF: Case Report Form
PA: Parathyroid autotransplantation
PTH: Parathyroid hormone
SPIRIT: Standard Protocol Items: Recommendations for Interventional Trials
T3: Triiodothyronine
T4: Thyroxine
TSH: Thyroid-stimulating hormone
