# Supplementary figures and images for: Parathyroid autotransplantation at a novel site for better evaluation of the grafted gland function: study protocol for a prospective, randomized controlled trial
Source: Trials. 2019 Jan 31;20:96. doi: 10.1186/s13063-019-3195-9 (PMC6357396; doi:10.1186/s13063-019-3195-9)

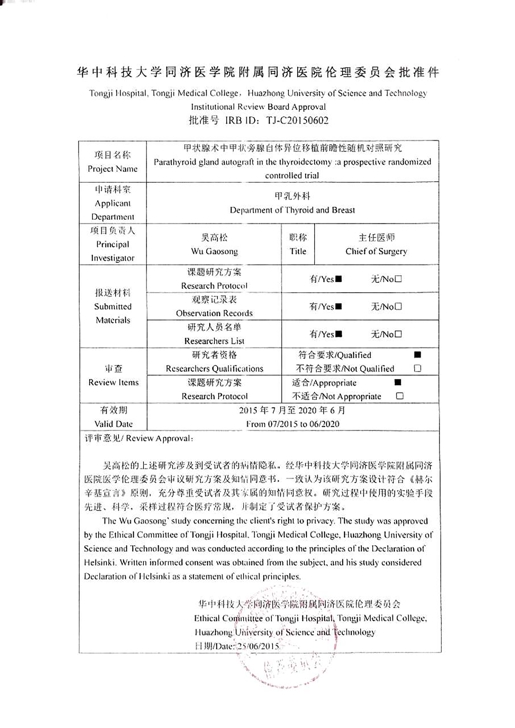

Supplement: Supplementary file 1 — Ethic certification of clinical trials. (JPG 184 kb) [file 13063_2019_3195_MOESM1_ESM.jpg]
